# Oral Microbial and Systemic Lipid Profiles in Patients With Asymptomatic Retained Partially Erupted Third Molars: A Preliminary Cross-Sectional Study

**DOI:** 10.1155/ijod/4770383

**Published:** 2025-11-20

**Authors:** Kamis Gaballah, Priyadharshini Sekar, Duaa Abuzayed, Aisha Alowais, Asia Shakir, Aya Razouk, Marwan Mansoor Mohammed

**Affiliations:** ^1^Department of Oral and Craniofacial Health Sciences, College of Dental Medicine, University of Sharjah, Sharjah, UAE; ^2^Microbiota Research Group, Research Institute for Medical and Health Sciences, University of Sharjah, Sharjah, UAE

**Keywords:** *Candida*, dyslipidemia, lipid profile, oral microbiome, systemic inflammation, third molars

## Abstract

**Background:**

Partially erupted (PE) third molars are frequently retained in the oral cavity and may contribute to localized inflammation. Chronic subclinical inflammation at these sites has been suggested to influence systemic markers such as lipid profiles, potentially increasing cardiovascular risk. Additionally, microbial colonization, particularly by bacteria and *Candida* species, may exacerbate inflammatory responses.

**Objective:**

To investigate the lipid panel parameters and oral microbial burden in individuals with PE third molars compared to those with fully erupted (FE) third molars and to assess potential associations with systemic inflammation.

**Methods:**

A cross-sectional study was conducted on 64 participants aged 20–35 years, divided into two groups: group 1 with PE third molars and group 2 with FE third molars. Fasting lipid profiles were measured using the CardioChek PA Analyzer. Gingival crevicular fluid (GCF) samples were collected from under the operculum in group 1 and from the gingival sulcus in group 2. Quantitative PCR (qPCR) was used to assess *Candida* and bacterial load. Statistical analysis included unpaired *t*-tests and multivariate logistic regression, adjusting for confounders.

**Results:**

A higher prevalence of elevated total cholesterol (TC) was observed in group 1 (15.63%) compared to group 2, with an odds ratio (OR) of 7.215 (95% CI: 0.992–52.5; *p*=0.051). *Candida* presence was significantly greater in PE sites (*p*=0.0036), though load differences were not statistically significant except in triglyceride (TG)-associated samples. All samples were positive for bacteria; however, bacterial load was unexpectedly higher in FE sites (*p*=0.0066). Associations between lipid abnormalities and microbial presence were noted but not statistically conclusive.

**Conclusion:**

PE third molars may be linked to elevated cholesterol levels and increased microbial colonization, particularly by *Candida* species. While no statistically significant associations were found, the trends suggest a potential inflammatory and metabolic role of third molar retention. Further longitudinal studies with larger sample sizes and detailed microbial profiling are needed to clarify these relationships.

## 1. Introduction

The third molars, commonly referred to as wisdom teeth, are the last teeth to erupt and are often retained in a partially erupted (PE) or impacted state. While third molars are frequently asymptomatic, their presence has been associated with localized periodontal inflammation, even in the absence of evident clinical symptoms [1]. Recent research suggests that chronic low-grade oral inflammation may contribute to systemic inflammatory burden, potentially influencing metabolic markers such as lipid profiles and increasing cardiovascular risk [2].

Systemic inflammation plays a pivotal role in the pathogenesis of atherosclerosis, with dyslipidemia serving as an important biomarker of early vascular dysfunction. Elevated levels of low-density lipoprotein (LDL), total cholesterol (TC), triglycerides (TGs), and unfavorable TC/high-density lipoprotein (HDL) ratios are known to correlate with endothelial damage and proinflammatory states [3–5]. The oral cavity, particularly sites harboring subclinical inflammation such as PE third molars, may serve as a persistent source of inflammatory stimuli [6, 7].

In addition to systemic markers, the microbial profile of the oral cavity may provide insight into the inflammatory potential of retained third molars [8, 9]. Colonization by pathogenic bacteria and fungi in these sites may exacerbate localized inflammation and contribute to systemic effects through microbial translocation or immune modulation. Qualitative and quantitative techniques now allow for the precise identification and quantification of key bacterial and fungal species associated with oral and systemic inflammation [10].

Recent studies have demonstrated a link between periodontal disease and hyperlipidemia, both of which are chronic inflammatory conditions that share common risk factors. The impact of periodontal inflammation on the development of atherosclerosis supports the hypothesis of a bidirectional relationship between periodontitis and hyperlipidemia. Elevated levels of proinflammatory cytokines are characteristic of both diseases. Furthermore, individuals with hyperlipidemia have shown positive responses to periodontal treatment, and the use of statins has been associated with improved periodontal health [11]. Hyperlipidemia also affects the trabecular and cortical structures of the mandible by inhibiting osteoblastic activity, increasing osteoclast numbers, and reducing bone mineral density [12].

Despite these theoretical links, evidence remains scarce regarding the systemic implications of asymptomatic partially or fully erupted (FE) third molars [13]. In particular, the impact of retained PE third molars on lipid metabolism and oral microbiota composition has not been fully elucidated.

This study aimed to assess the lipid and microbial profiles of individuals with retained PE third molars, comparing those with FE versus PE third molars, to evaluate their potential association with systemic inflammation.

## 2. Materials and Methods

### 2.1. Study Design and Participant Selection

This exploratory study included 64 participants, aiming to assess differences in lipid profile parameters—namely, TC, HDL, calculated LDL, and the TC/HDL ratio—using the CardioChek PA Analyzer (Polymer Technology Systems, Inc., Indianapolis, USA). Participants were required to fast for 9–12 h prior to capillary blood collection via finger prick for lipid profiling.

Participants were randomly selected and aged between 20 and 35 years, with the inclusion criteria being healthy (ASA1) and having normal or overweight BMI. Exclusion criteria included the presence of chronic systemic diseases (e.g., diabetes and hypertension), obese, a history of hyperlipidemia, gingival inflammation, and pericoronitis. The study investigated the potential association between dyslipidemia and the presence of PE third molars.

Participants were categorized into two groups:

  Group 1: Individuals with FE third molars, which served as a control group.
  Group 2: Individuals with PE third molars, which served as a test group.

### 2.2. Gingival Crevicular Fluid (GCF) Sampling

GCF samples were collected to assess microbial load. For the test group (patients with PE third molars), samples were obtained from beneath the operculum covering the PE molars using sterile paper points. For the control group (patients with FE third molars), GCF samples were collected from the gingival sulcus of the corresponding third molars.

All samples were collected in the morning to minimize diurnal variation. Prior to sampling, the selected sites were isolated with cotton rolls and gently air-dried to prevent contamination with saliva. Paper points were inserted into the gingival crevice for 30 s, then immediately transferred into sterile microcentrifuge tubes and stored at –80°C until RNA extraction and further analysis.

### 2.3. Real-Time Quantitative PCR (qPCR)

Real-time qPCR was employed to quantify the microbial load of *Candida* species and bacteria, targeting the CALB gene and 16S rRNA, respectively, using primers described in previous studies [14, 15]. Total RNA was extracted from GCF samples using the RNeasy Mini Kit (Cat. No. 74104, Qiagen), following the manufacturer's instructions. Reverse transcription into complementary DNA (cDNA) was performed using the QuantiTect Reverse Transcription Kit (Cat. No. 205311, Qiagen).

qPCR amplification was carried out using the HOT FirePol EvaGreen qPCR Supermix (Cat. No. 08-36-00001) in 20 µL reactions, adhering to the manufacturer's recommended concentrations and cycling parameters. Amplification was performed on the QuantStudio 3 Real-Time PCR System, and results were reported as threshold cycle (Ct) values.

### 2.4. Statistical Analysis

Data analysis was performed using SPSS version 29 (SPSS Inc., Chicago, USA). Continuous variables were expressed as means.

Statistical comparisons of lipid profiles between groups were conducted using unpaired *t*-tests (GraphPad Prism version 5, Boston, MA, USA). Logistic regression models were used to analyze associations between third molar status and dyslipidemia, adjusting for potential confounders including age, gender, smoking status, systemic disease, and third molar laterality. A *p*-value of <0.05 was considered statistically significant.

### 2.5. Ethical Considerations

The study was reviewed and approved by the University of Sharjah Research Ethics Committee (Approval REC-23-04-14-01-S). Written informed consent was obtained from all participants prior to their involvement in the study. All participants were adults; therefore, no consent from parents or guardians was required.

## 3. Results

A sample of 64 participants were examined for this study with fair distribution between genders in both groups. The age ranged from 18 to 35 years old with a mean of 23.47 for group 1 and 22.66 for group 2 (Table 1).

**Figure 1:**
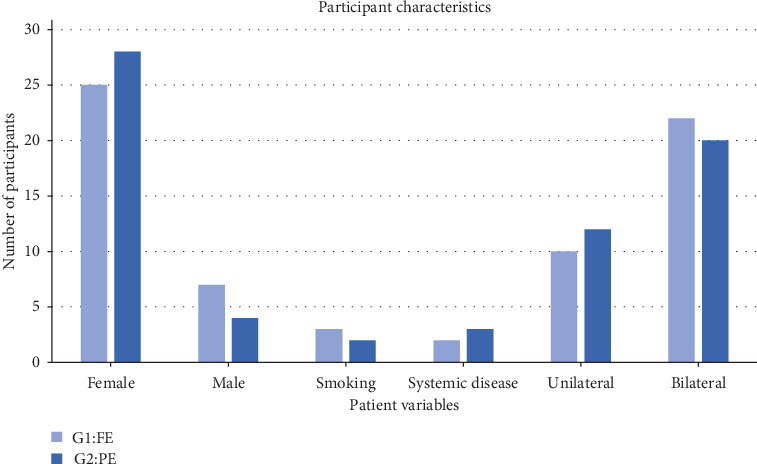
Descriptive participants characteristics in the fully erupted and partially erupted third molar groups.

The majority of the participants were medically fit with a fair distribution between the two groups, and a total of 7.81% of participants were affected with systematic diseases. A value of 7.81% of the participants reported smoking or history of smoking cigarettes, vape, or waterpipe. The distribution of types of smoking and in between groups is fair. The participant had a similar percentage of bilateral/unilateral teeth distributed fairly among the groups (Figure 1).

**Figure 2:**
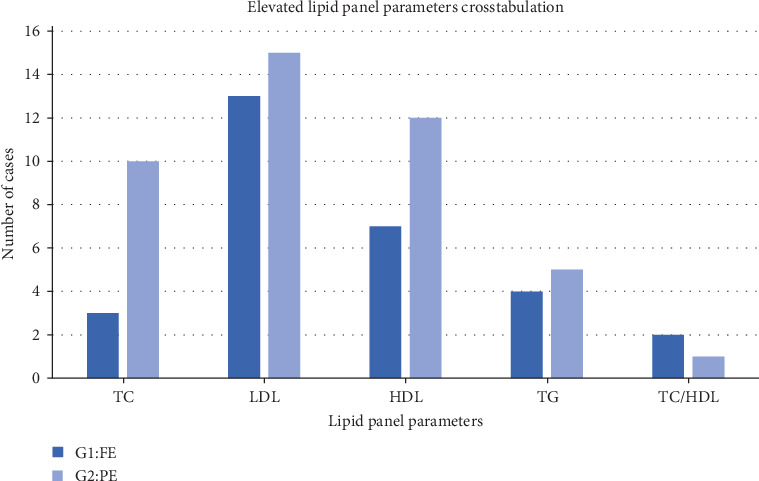
Number of cases with elevated lipid panel parameters in the fully erupted and partially erupted third molar groups.

According to the American Heart Association classification of lipid panel parameters, the optimal values are as follows: TC (below 200 mg/dL), HDL cholesterol (above 60 mg/dL), LDL cholesterol (below 100 mg/dL), and TGs (below 150 mg/dL) [9]. The PE group showed more than three folds increase (15.63%) in the prevalence of high cholesterol when compared with the FE group. The LDL value was high in both groups (43.75%), with fair distribution between groups. The prevalence of high HDL was almost two folds higher in group 2 (18.75%) in comparison with group 1. The prevalence of high TGs was 14.1% with fair distribution between groups (Figure 2).

In multivariate logistic regression (Table 2) adjusted for age, gender, smoking status, laterality, and presence of systematic disease, no relevant associations were observed between third molars and lipid markers. Association between PE third molars and elevated TC levels was 7.215 times higher than FE third molars (odds ratio [OR]: 7.215, 95% CI, 0.992 – 52.5; *p*=0.051); the association is statistically insignificant.

### 3.1. Presence and Load of *Candida* and Bacteria

**Figure 3:**
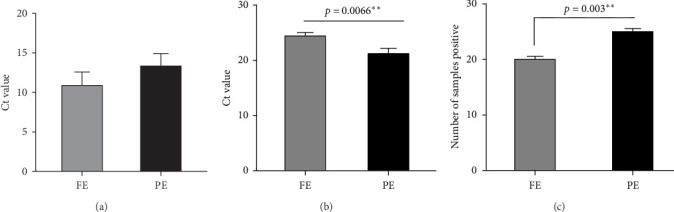
(A) Candidal load in FE and PE, (B) bacterial load in FE and PE, and (C) candidal presence in FE and PE. *⁣*^*∗∗*^ indicate a statistically significant difference.

A total of 20 samples were positive for *Candida* in FE and 25 samples were positive for *Candida* in PE. The number of samples positive for Candidal presence in PE was significantly higher (*p*-value = 0.0036). All the samples in both FE and PE were positive for bacteria. The *Candida* and bacterial load were determined by Ct value. The mean ± SD Ct value of *Candida* present in FE and PE was 10.90 ± 9.578 and 13.34 ± 8.981, respectively. There was no statistical difference in the candidal load between FE and PE. The mean ± SD Ct value of bacteria present in FE and PE was 24.45 ± 3.515 and 21.22 ± 5.452, respectively. Bacteria was significantly more present on FE compared to PE with a *p*-value of 0.0066 as seen in Figure 3.

### 3.2. Comparison of Lipid Profile and Candidal Load in Both Groups

**Figure 4:**
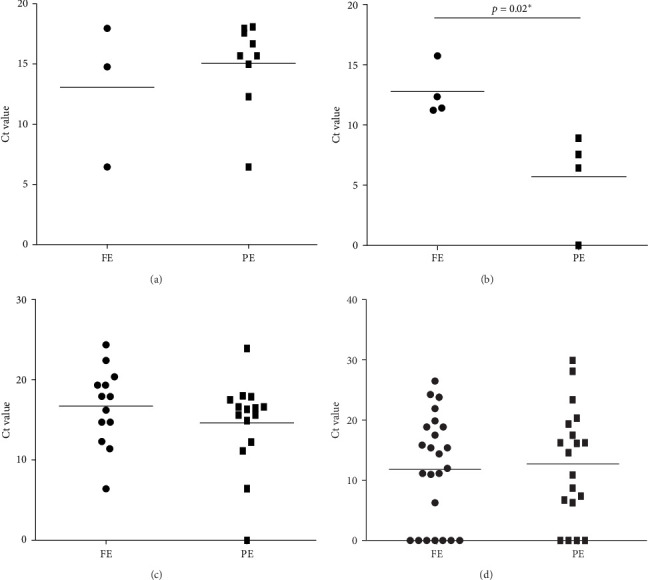
Comparison of Ct value of *Candida* (load) with (A) high TC, (B) high triglyceride, (C) high LDL, and (D) low LDL, in FE and PE. *⁣*^*∗*^ indicates a statistically significant difference.

A total of three and nine samples that were positive for *Candida* and associated with high TC in FE and PE, respectively, were observed. Candida cultivation was significantly reported more in PE when compared to FE (*p*-value = 0.0018). But the *Candida* load in high TC samples in both FE and PE was comparable with a mean ± SD Ct value of 13.07 ± 5.945 and 15.05 ± 3.695, respectively. Equal number of samples with high TGs was positive for *Candida* in both FE and PE (*n* = 4). However, the *Candida* load was statistically higher in FE (*p*-value = 0.02) when compared to PE. Their mean ± SD Ct value was 12.73 ± 2.104 and 5.746 ± 3.962, respectively. There was no comparable difference in both high and low LDL and *Candida* presence and load in CE and PE as seen in Figure 4.

### 3.3. Comparison of Lipid Profile and Bacterial Load and Presence in CE and PE

**Figure 5:**
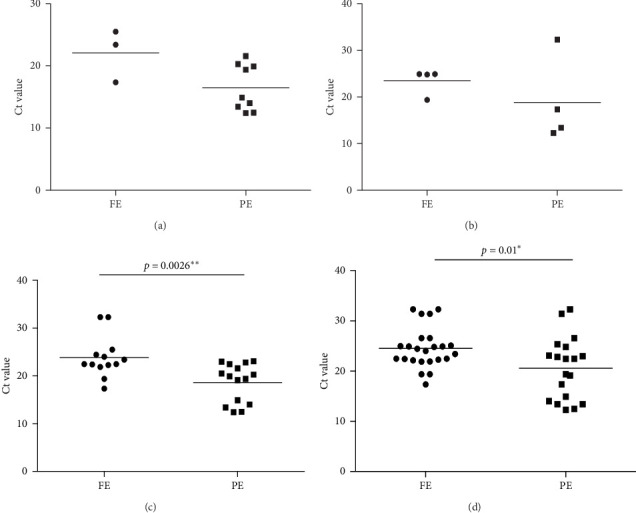
Comparison of Ct value of bacteria (load) with (A) high TC, (B) high triglyceride, (C) high LDL, and (D) low LDL, in FE and PE. *⁣*^*∗*^ and *⁣*^*∗∗*^ indicate a statistically significant difference.

A total of three and nine samples that were positive for bacteria and associated with high FC in CE and PE, respectively, were observed. Bacteria were significantly present more in PE when compared to FE (*p*-value 0.0018). The bacterial load, that is, Ct value in both FE and PE for samples high in TC and TGs, was comparable. There was no comparable difference in both high and low LDL and bacterial presence in FE and PE. However, the bacterial load was statistically higher in FE compared to PE among high LDL and low LDL samples (*p*-value 0.0026 and 0.01, respectively). The mean ± SD Ct value of bacterial load in FE and PE among high LDL samples was 23.88 ± 4.294 and 18.63 ± 4.031, respectively. The mean ± SD Ct value of bacterial load in FE and PE among low LDL samples was 24.54 ± 4.006 and 20.57 ± 6.158, respectively, as seen in Figure 5.

## 4. Discussion

This preliminary cross-sectional study aimed to investigate the potential relationship between PE third molars and systemic lipid profiles, as well as to characterize the microbial burden—specifically *Candida* species and total bacterial load—at the site of eruption. Although the logistic regression analysis did not yield statistically significant associations between third molar eruption status and abnormal lipid values, notable trends emerged. Most prominently, individuals with PE third molars exhibited a more than threefold increase in the prevalence of elevated TC compared to those with FE molars. These findings suggest a potential link between local oral inflammatory conditions and systemic metabolic alterations, warranting further investigation.

Previous research has supported the notion that retained third molars, even in the absence of overt symptoms, may act as chronic inflammatory foci. Offenbacher et al. [6] demonstrated increased probing depths and elevated systemic inflammatory markers—including serum IL-6, CRP, and sICAM—in individuals with retained third molars. The localized inflammation observed in these cases is thought to trigger systemic immune responses, possibly by promoting the release of proinflammatory cytokines and mediators into the circulation [13, 16, 17]. Our findings of elevated TC in individuals with PE molars may reflect this systemic inflammatory state, although causality cannot be confirmed in the present cross-sectional design. Extraction of 3^rd^ molar might reduce the inflammation and prevent further spread as it acts as chronic source of inflammation and infection [18].

The relation between impacted or semi-impacted third molars and increased systemic inflammation has been investigated by Graziani et al. [16], and they revealed significantly higher TG levels in patients with semi-impacted 3^rd^ molars compared to those who do not have 3^rd^ molars. Later they compared semi-impacted 3^rd^ molars before and after removal [16]. At baseline, increased levels of systemic inflammation and oxidative stress and decreased lipid fractions were observed in the group indicated for extraction in comparison to the control group, with no changes in lipid profile. After 3 months, the investigators noticed a reduction in the C-reactive proteins, fibrinogen, and plasma lipoperoxides in the extraction group compared to the control group with no changes in lipid profile. However, the endothelial function remained unaffected by the extraction. The study concluded that the extraction of third molars is associated with a transient systemic inflammatory response of 1-week duration, followed by a reduction in oxidative stress 3 months later. The extraction of third molars to reduce associated systemic inflammation might bring beneficial effects; however, further investigations are required [16].

Notably, the relationship between lipid metabolism and chronic inflammation is well documented. Dyslipidemia is not only a marker but also a driver of systemic inflammation, particularly in the context of atherosclerosis and cardiovascular disease (CVD). Elevated TC and LDL levels promote endothelial dysfunction, leukocyte activation, and increased expression of adhesion molecules, all of which are hallmarks of a chronic inflammatory state [19]. This shared inflammatory pathway between periodontal and lipid disorders has led to the proposal of a bidirectional relationship between periodontitis and hyperlipidemia [5, 11].

While the observed OR (7.215) for elevated TC in the PE group did not reach statistical significance (*p*=0.051), the strength of the association suggests that this finding may have clinical relevance. It is possible that the limited sample size in this study underpowered the regression analysis, and a larger cohort may yield significant results.

The observed lipid profile in the PE group—characterized by a higher prevalence of elevated TC coupled with a lower prevalence of optimal HDL levels compared to the FE group—presents a seemingly paradoxical yet clinically significant pattern. This specific dyslipidemic phenotype (high TC/low HDL) is a well-established risk factor for CVD and is often driven by a state of chronic inflammation [17]. We hypothesize that the localized, subclinical inflammation associated with PE third molars acts as a persistent immune stimulus. This chronic inflammatory state can lead to hepatic dysregulation, promoting the synthesis of apolipoprotein *B*-containing lipoproteins (such as LDL and VLDL, which contribute to TC) while simultaneously suppressing the production of apolipoprotein A-I, the primary protein component of HDL [17, 20]. Furthermore, systemic inflammation can increase the activity of enzymes like cholesteryl ester transfer protein (CETP), which facilitates the exchange of cholesteryl esters from HDL for TGs from LDL and VLDL, resulting in TG-rich, cholesterol-poor HDL particles that are rapidly cleared from the circulation. This mechanism would explain the concurrent rise in atherogenic cholesterol and decline in cardioprotective HDL, effectively linking the local oral inflammatory niche to a systemic metabolic dysregulation that favors atherogenesis.

In contrast to the previous study, Kindler et al. [13] investigated the association between systemic inflammation and impacted, erupted, or nonexistent third molars. They revealed that neither impacted nor erupted teeth were associated with high-sensitivity C-reactive protein, white blood cell count, lipid profile, or fibrinogen as markers for systemic inflammation. The erupted third molars group had markedly lower serum concentrations of leptin, Angiopoietin-2, and Ang- 2/TIE ratio compared to the nonexistent third molars group [13]. No such associations were observed for the impacted third molars group. The following evidence did not corroborate an association between third molars and an increase in systemic inflammatory markers. Thus, extraction of third molars should be done selectively, especially in medically compromised patients [13].

Findings from our study showed that 43.75% of the participants had high LDL cholesterol levels, which can be attributed to the population as the vast majority of participants are university students. Other studies reported similar elevations in lipid profile among university students. Al-Ajlan [21] studied the relationship between anthropometric measurements and lipid profile among male Saudi university students between 18 and 35 years; the results showed a positive correlation between BMI and TC and LDL. In Brazil, elevated TC, LDL, and TG in university students were attributed to sedentary lifestyle and smoking [20].

The microbial data reveal significantly higher *Candida* presence in the GCF of participants with PE third molars compared to FE controls. In a study by Campbell et al. [10], the increased abundance of *Veillonella* in convergent impactions, and distinctive bacterial profiles at horizontal impactions were observed, which may be implicated in the association between convergent angles and caries. The subgingival operculum overlying PE molars provides a sheltered, anaerobic environment conducive to microbial accumulation and biofilm development. These niches are notoriously difficult to clean, leading to persistent microbial colonization and localized inflammation [22].

Although *Candida* presence was significantly higher in the PE group, no significant difference in *Candida* load (as determined by Ct values) was observed, except in samples associated with elevated TGs, where, unexpectedly, higher *Candida* load was found in the control group. This suggests that while *Candida* colonization is more common in PE third molars, the burden of infection may be modulated by other host or microbial factors.

Bacterial presence was universal across both groups, as expected. However, bacterial load was significantly higher in the control group, especially among samples associated with high LDL and low LDL levels. This finding is somewhat counterintuitive, as one would expect increased bacterial accumulation in the more anatomically compromised and inflamed sites. One possible explanation is that the bacterial species composition differs between groups. Sites with PE molars may harbor more virulent, but less abundant, pathogens such as *P. gingivalis* or *T. forsythia*, while FE molars may have greater biomass but less pathogenic species. Unfortunately, species-level identification was not performed in this study. Future studies using 16S rRNA sequencing or metagenomics would provide valuable insight into the compositional differences between these biofilms.

Moreover, the role of oral microorganisms in modulating systemic lipid metabolism has gained increasing attention. Several bacterial species associated with periodontal disease have been shown to induce hepatic lipid synthesis through toll-like receptor (TLR)-mediated pathways [23]. This is supported by findings from other studies, which have demonstrated that *P. gingivalis* plays a central role in linking periodontal disease to CVD through its potent ability to modify apolipoproteins and enhance oxidative stress, thereby contributing to vascular inflammation and atherogenesis [24, 25]. Similarly, systemic exposure to *Candida albicans* can alter lipid homeostasis and immune function, particularly in immunocompromised or metabolically dysregulated hosts [26].

This study has several limitations. First, its cross-sectional design prevents causal inferences between third molar status, lipid profiles, and microbial colonization. Second, the relatively small and homogenous sample of young university students may limit statistical power and generalizability. Third, while our inclusion and exclusion criteria minimized some confounding factors, residual confounders such as dietary habits, physical activity, and body composition could not be fully controlled. Fourth, microbial analysis was limited to overall *Candida* and bacterial load, without species-level identification. Finally, the absence of a third comparator group (unerupted or previously extracted third molars) restricts interpretation of whether third molar retention itself is directly responsible for systemic alterations.

Future research should therefore adopt a longitudinal design, incorporating larger and more diverse populations, a priori power calculations, and additional groups such as individuals without third molars or those undergoing extractions. Monitoring systemic biomarkers—including inflammatory mediators and detailed lipid fractions—before and after third molar removal would provide stronger evidence of causal relationships. Incorporating advanced microbial profiling techniques would also clarify the compositional and functional role of oral biofilms in modulating systemic lipid metabolism.

## 5. Conclusion

In summary, this study identified higher rates of hypercholesterolemia and increased microbial colonization—especially *Candida*—in individuals with PE third molars compared to those with FE molars. Although statistical significance was not achieved for lipid associations, the trends observed are consistent with existing literature on oral-systemic inflammatory pathways. Further research with larger samples, longitudinal design, and detailed microbial profiling is warranted to clarify these associations and inform clinical decision-making regarding third molar management.

## Figures and Tables

**Table 1:** Mean ± standard deviation values of study parameters in the fully erupted and partially erupted third molar groups.

Group	Age (years)	TC	LDL	HDL	TG	TC/HDL ratio
Group 1 FE						
Mean Standard deviation	23.47	164.94	92.13	54.28	96.25	3.14
	3.05	32.19	32.69	11.10	45.33	0.87
Group 2 PE						
Mean Standard deviation	22.66	175.72	97.25	57.00	107.78	3.23
	0.90	36.26	35.90	12.69	66.05	1.06

**Table 2:** Multivariate logistic regression model for relation between fully erupted and partially erupted third molars and elevated lipid parameters.

Lipid panel	Adjusted odds ratio		
	a(OR)	a(OR) 95% CI	a(OR) *p*-Value
TC high	7.215	0.992 – 52.5	0.051
LDL high	0.745	0.188 – 2.963	0.676
HDL low	0.676	0.179 – 2.551	0.563
TG high	1.888	0.402 – 8.878	0.421
TC/HLR ratio high	0.166	0.008 – 3.493	0.248

## Data Availability

The data that support the findings of this study are available from the corresponding author upon reasonable request.
